# Influenza incidence overlapped with COVID‐19 or under COVID‐19 control measures

**DOI:** 10.1002/iid3.672

**Published:** 2022-07-12

**Authors:** Azad Shokri, Ghobad Moradi, Farhad Moradpour, Amjad Mohamadi Bolbanabad, Fatemeh Younesi, Parisa Daftarifard, Ali Ebrazeh

**Affiliations:** ^1^ Social Determinants of Health Research Center, Research Institute for Health Development Kurdistan University of Medical Sciences Sanandaj Iran; ^2^ Department of Health Services Management, Faculty of Management and Information Iran University of Medical Sciences Tehran Iran; ^3^ English Language Research Center, South Tehran Branch IAU Tehran Iran; ^4^ Department of Public Health, School of Public Health Qom University of Medical Sciences Qom Iran

**Keywords:** COVID‐19, influenza, nonpharmaceutical interventions

## Abstract

**Introduction:**

COVID‐19 pandemic caused infection when influenza was still prevalent. This study was conducted to examine influenza incidence overlapped with COVID‐19 and the effect of the COVID‐19 measures on influenza incidence as a proxy.

**Methods:**

The routine sentinel surveillance data on COVID‐19 and influenza was obtained from the national integrated care electronic health record system. Data were collected in 28 points from 11 months before the outbreak (from March 2019 to January 2020) and 17 months after the outbreak (February 2020 to June 2021).

**Results:**

In Iran, the incidence rate of influenza was 51.1 cases per 100,000 populations in November 2019, while it was only 0.1 in November 2020. The average number of influenza cases specifically for the Kurdistan province during the seasonal flu peak in 2019–2020 was 2.5 cases per 100,000 populations, while the average of influenza cases in the preceding 4 years was 0.4 cases per 100,000 populations. In other words, the seasonal peak of influenza in Iran was significantly higher than that of previous and after years.

**Conclusion:**

It seems that some of the nonpharmaceutical interventions (NPIs) used to control COVID‐19 are effective against influenza epidemics and the results indicated a marked decline in the number of influenza cases may cause after the implementation of public health measures for COVID‐19. The results showed the seasonal peak of influenza in Iran was significantly higher than that of previous years, so it seems that the influenza winter peak season (November 2019) overlapped with SARS‐CoV‐2 causing observed undetected infection during influenza winter peak.

## Graphical abstract

To examine the effect of the COVID‐19 measures on influenza and overlap it with COVID‐19, routine sentinel surveillance data on COVID‐19 and influenza illnesses for Iran. It seems that some of the NPIs used to control COVID‐19 have been shown to be effective against influenza epidemics. However the results indicated a marked decline in number of influenza cases may cause after the implementation of public health measures for COVID‐19, it seems that influenza winter peak season (November 2019) overlapped with SARS‐CoV‐2 cause observed undetected infection during influenza winter peak.

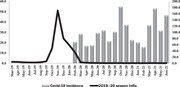

## 1 INTRODUCTION

The coronavirus disease 2019 (COVID‐19) pandemic has spread across the world with 539 million cases and 6 million deaths on June 11, 2022.^1^, ^2^ COVID‐19 pandemic caused infection when influenza is still prevalent. It seems that public health efforts to manage COVID‐19 most likely reduced influenza transmission in early 2020. Influenza is known to be a worldwide public health priority, affecting about 5%–10% of all countries every year, with an estimated number of 250,000–500,000 deaths globally.^3^ The epidemiology and clinical features of COVID‐19 and influenza are common. The influenza virus has comparable transmission traits with COVID‐19, which includes direct contact and transmission via airborne droplets.^4^ Both diseases share common symptoms and clinical features including fever, cough, rhinitis, sore throat, headache, dyspnea, and myalgia. However, acute respiratory distress syndrome (ARDS) is much less in influenza and mortality is <1%, while at the same time as ARDS is more common and mortality is 3%–4% in COVID‐19. Consequently, the COVID‐19 pandemic and the implementation of nonpharmaceutical interventions (NPIs; e.g., cessation of global travel, wear of gloves, mask use, physical distancing, and staying home) decreased the transmission of a few viral respiratory pathogens such as Influenza.^5^ A pattern seen in the United States was that the number of influenza patients for the 2019–2020 seasons reducing earlier than expected. Data from medical laboratories in the United States indicated a 61% reduction in the number of specimens reported and a 98% reduction in influenza activities as measured by the percentage of reported specimens testing positive (from a mean of 19.34% to 0.33%). Influenza data reported by WHO from three Southern Hemisphere countries including Oceania (Australia), South America (Chile), and Southern Africa (South Africa) confirmed very low influenza activity during the months that represent the typical Southern Hemisphere influenza season. Implementation of NPIs in periods of increased transmission could substantially decrease the rates of infection for each disease.^6^ Therefore, to perceive the overall potential decrease in respiratory virus transmission, we examined influenza incidence overlapped with COVID‐19 and the effect of the COVID‐19 measures on influenza incidence as a proxy.

## 2 METHODS

We obtained routine hospital sentinel surveillance data on outpatient and hospitalization with COVID‐19 and influenza‐related to seven provinces from a national integrated care electronic health record system.^7^ COVID‐19 cases were confirmed by laboratory tests. Influenza cases include the number of patients with influenza‐like illnesses (ILIs) and acute respiratory infections 15%–20% of them are sent to the national reference laboratory and are confirmed tested for influenza viruses. The data of seven provinces were collected in 28 points from 11 months before the outbreak (from March 2019 to January 2020) and 17 months after the outbreak from February 2020 to June 2021). Weekly seasonal flue incidence between 2020 and 2016 was collected only for one province of Iran, due to limited access to data.

## 3 RESULTS AND DISCUSSION

**Figure 1:**
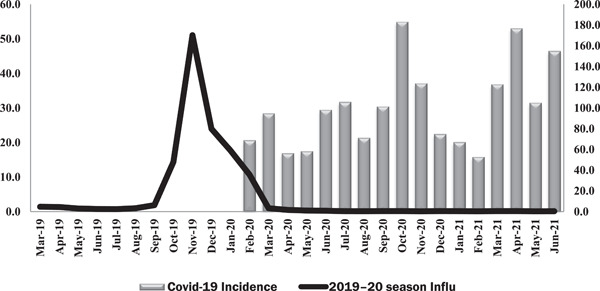
Trend of the monthly influenza cases before and after the onset of COVID‐19 (March 2019 to June 2021) per 100,000 population

Figure 1 shows the monthly incidence of influenza and COVID‐19 since 2019., the incidence of influenza has been raised since September 2019 and reached its peak in November 2019. after then, along with the onset of COVID‐19, it began to decline sharply. In other words, the incidence of influenza in November 2019 was 51.1 cases per 100,000 populations, while in November 2020 only 0.1 cases per 100,000 populations were reported.

However, when SARS‐CoV‐2 was in circulation whether the ability to detect influenza was compromised (e.g., primary healthcare facilities being closed to patients with “normal” seasonal influenza), or whether, by counting more serious diseases (e.g., hospitalized patients), it seems that some of the NPIs used to control COVID‐19 be effective against influenza circulation. Similar to the present study, in China, the mean incidence rate of influenza was reduced by 64% after the implementation of NPIs to prevent COVID‐19.^8^ In Singapore, the incidence of influenza in 5–9 epidemiologic weeks in 2020 has decreased by 64% and the daily rate of influenza by 76% compared with the 3 previous epidemiologic years between 2016 and 2019.^9^ Influenza activity in the United States also increased in early November 2019, while the inter‐seasonal influenza periods in the United States reached their lowest rates in recent years in 2020. Influenza has also been reduced in the Southern Hemisphere countries including Australia, Chile, and South Africa.^10^

**Figure 2:**
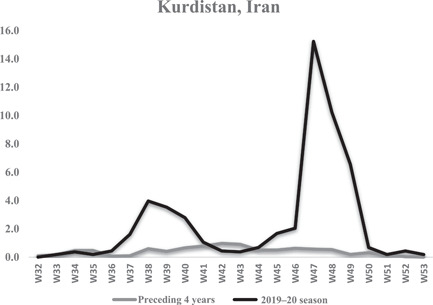
Weekly seasonal flue incidence between 2020 and 2019 compared to the average weekly seasonal flu incidence between 2019 and 2016 per 100,000 population of Kurdistan province

Figure 2 shows the weekly seasonal influenza incidence between 2019 and 2020 compared to the average weekly seasonal influenza incidence between 2016 and 2019 in Kurdistan province as a sample of Iran provinces. The average influenza incidence specifically for the Kurdistan province during the seasonal influenza peak year 2019–2020 was 2.5 cases per 100,000 populations, while the average in of influenza cases in the preceding 4 years was 0.4 cases per 100,000 populations. This shows that the seasonal peak of influenza is significantly higher than that in previous years.

**Figure 3:**
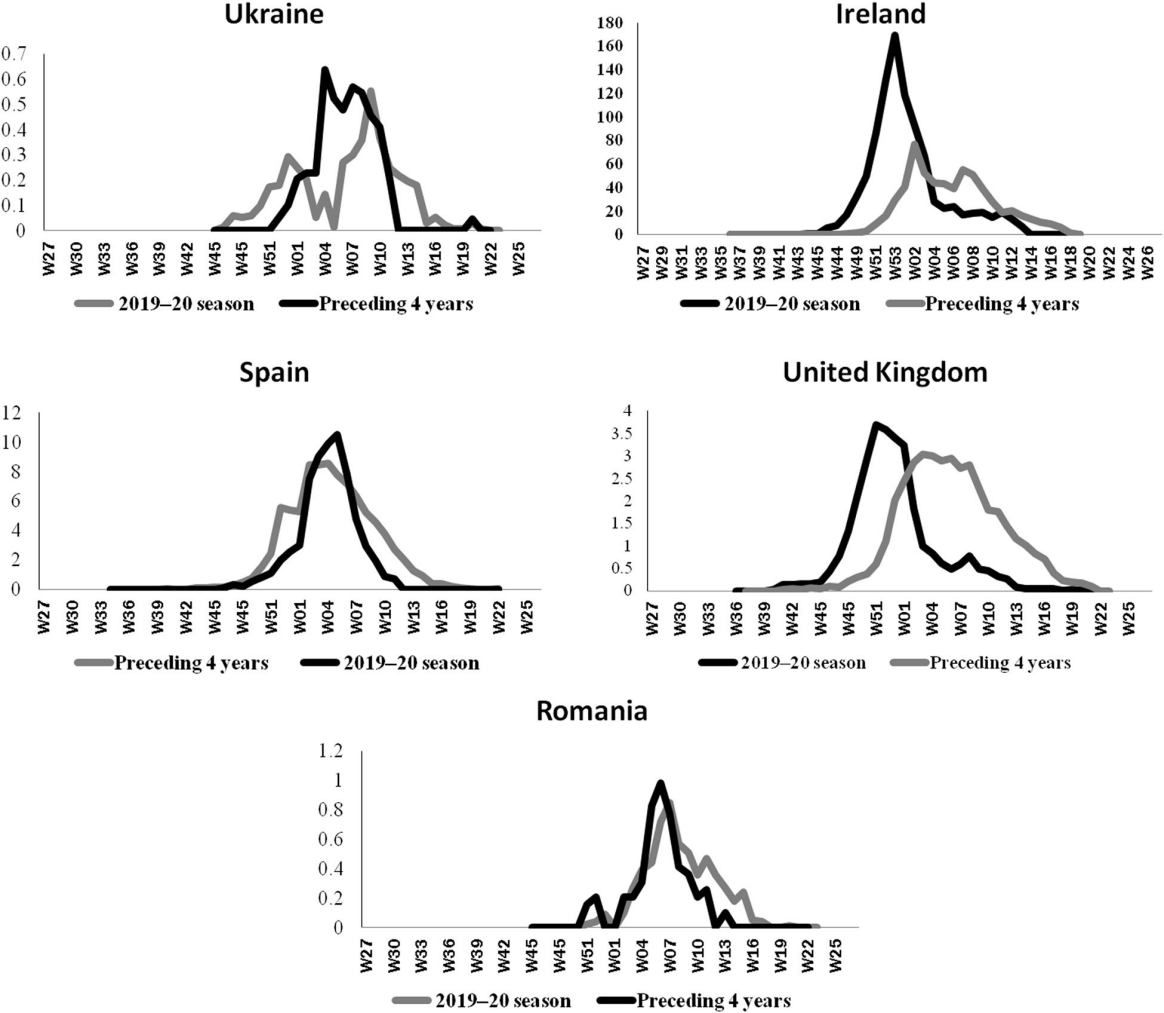
Weekly seasonal flue incidence between 2020 and 2019 compared to the average weekly seasonal flu incidence between 2019 and 2016 per 1 million population

The hospitalized laboratory‐confirmed influenza cases (ICU and other wards), in some European countries including Ireland, Ukraine, England, Spain, and Romania (https://flunewseurope.org/HospitalData) was showed that the seasonal peak of influenza in the epidemiological year 2019–2020 significantly higher than that of previous years (Figure 3). Furthermore, as the graphs indicate, an increase of 66%, 26%, 14%, 6%, and 4% of influenza incidence in 6–9 weeks during the seasonal influenza peak in the mentioned countries compared with the average incidence of seasonal influenza in the same period 4 years ago between 2016 and 2019.^11^

The results showed the seasonal peak of influenza in Iran was significantly higher than that of previous years, so it seems that the influenza winter peak season (November 2019) overlapped with SARS‐CoV‐2 causing observed undetected infection during the influenza winter peak. A study performed as a retrospective analysis of 1001 influenza patients in Wuhan, China, confirmed the co‐infection of the SARS‐CoV‐2.^12^

## AUTHOR CONTRIBUTIONS

Azad Shokri and Ali Ebrazeh contributed to the study conception, and Amjad Mohamadi Bolbanabad, Fatemeh Younesi, and Farhad Moradpour gathered data design, and all authors were involved in the data analysis and interpretation process. All authors drafted and translated the manuscript and all the authors contributed by their comments of improvement in several revisions to reach a final manuscript.

## CONFLICT OF INTEREST

The authors declare no conflicts of interest.

## References

[1] Worldometer. Reported Cases and Deaths by Country or Territory; 2021. Accessed August 15, 2021. https://www.worldometers.info/coronavirus/

[2] Dong E , Du H , Gardner L . An interactive web‐based dashboard to track COVID‐19 in real‐time. Lancet Infect Dis. 2020;20:533‐534. 32087114 10.1016/S1473-3099(20)30120-1 PMC7159018

[3] Di Giuseppe G , Pelullo CP , Paolantonio A , Della Polla G , Pavia M . Healthcare workers' willingness to receive influenza vaccination in the context of the COVID‐19 pandemic: a survey in Southern Italy. Vaccines. 2021;9:766. 34358182 10.3390/vaccines9070766 PMC8310353

[4] Chotpitayasunondh T , Fischer TK , Heraud JM , et al. Influenza and COVID‐19: what does co‐existence mean? Influenza Other Respir Viruses. 2021;15:407‐412. 33128444 10.1111/irv.12824 PMC8051702

[5] Olsen SJ , Winn AK , Budd AP , et al. Changes in influenza and other respiratory virus activity during the COVID‐19 pandemic—United States, 2020‐2021. Morb Mortal Wkly Rep. 2021;70:1013. 10.15585/mmwr.mm7029a1 PMC8297694 34292924

[6] Solomon DA , Sherman AC , Kanjilal S . Influenza in the COVID‐19 era. JAMA. 2020;324:1342‐1343. 32797145 10.1001/jama.2020.14661

[7] Riazi H , Jafarpour M , Bitaraf E . Towards National eHealth Implementation—a Comparative Study on WHO/ITU National eHealth Strategy Toolkit In Iran. Stud Health Technol Inform. 2014;205:246‐250. 25160183

[8] Lei H , Xu M , Wang X , et al. Nonpharmaceutical interventions used to control COVID‐19 reduced seasonal influenza transmission in China. J Infect Dis. 2020;222:1780‐1783. 32898256 10.1093/infdis/jiaa570 PMC7499620

[9] Soo RJJ , Chiew CJ , Ma S , Pung R , Lee V . Decreased influenza incidence under COVID‐19 control measures, Singapore. Emerging Infect Dis. 2020;26:1933‐1935. 10.3201/eid2608.201229 PMC7392467 32339092

[10] Olsen SJ , Azziz‐Baumgartner E , Budd AP , et al. Decreased influenza activity during the COVID‐19 pandemic—United States, Australia, Chile, and South Africa, 2020. Am J Transplant. 2020;20:3681‐3685. 33264506 10.1111/ajt.16381 PMC7753605

[11] European Centre for Disease Prevention and Control (ECDC) and World Health Organization Regional Office for Europe. (WHO/Europe). Weekly Influenza Overview. Accessed May 23, 2022. https://flunewseurope.org/

[12] Zheng X , Wang H , Su Z , et al. Co‐infection of SARS‐CoV‐2 and influenza virus in early stage of the COVID‐19 epidemic in Wuhan, China. J Infect. 2020;81:e128. 32474045 10.1016/j.jinf.2020.05.041 PMC7255712

